# Sphingolipids as Modulators of SARS-CoV-2 Infection

**DOI:** 10.3389/fcell.2021.689854

**Published:** 2021-06-17

**Authors:** Kid Törnquist, Muhammad Yasir Asghar, Vignesh Srinivasan, Laura Korhonen, Dan Lindholm

**Affiliations:** ^1^Minerva Foundation Institute for Medical Research, Helsinki, Finland; ^2^Faculty of Science and Engineering, Cell Biology, Åbo Akademi University, Turku, Finland; ^3^Medicum, Department of Biochemistry and Developmental Biology, Faculty of Medicine, University of Helsinki, Helsinki, Finland; ^4^Department of Child and Adolescent Psychiatry and Department of Biomedical and Clinical Sciences, Linköping University, Linköping, Sweden

**Keywords:** SARS-CoV-2, sphingosine, ceramide, drugs, COVID-19, biomarker

## Abstract

Severe acute respiratory syndrome coronavirus 2 (SARS-CoV-2) is the cause of the COVID-19 pandemic with severe consequences for afflicted individuals and the society as a whole. The biology and infectivity of the virus has been intensively studied in order to gain a better understanding of the molecular basis of virus-host cell interactions during infection. It is known that SARS-CoV-2 binds to angiotensin-converting enzyme 2 (ACE2) via its spike protein. Priming of the virus by specific proteases leads to viral entry via endocytosis and to the subsequent steps in the life cycle of SARS-CoV-2. Sphingosine and ceramide belong to the sphingolipid family and are abundantly present in cell membranes. These lipids were recently shown to interfere with the uptake of virus particles of SARS-CoV-2 into epithelial cell lines and primary human nasal cells in culture. The mechanisms of action were partly different, as sphingosine blocked, whilst ceramide facilitated viral entry. Acid sphingomyelinase (ASM) is vital for the generation of ceramide and functional inhibition of ASM by drugs like amitriptyline reduced SARS-CoV-2 entry into the epithelial cells. Recent data indicates that serum level of sphingosine-1-phosphate (S1P) is a prognostic factor for COVID-2 severity. Further, stimulation of sphingosine-1-phosphate receptor 1 (S1PR1) might also constrain the hyper-inflammatory conditions linked to SARS-CoV-2. Here, we review recent exciting findings regarding sphingolipids in the uptake of SARS-CoV-2 and in the course of COVID-19 disease. More studies are required on the mechanisms of action and the potential use of antidepressant drugs and sphingolipid modifiers in SARS-CoV-2 infections and in the treatment of the more serious and fatal consequences of the disease.

## Introduction

A great deal of effort is currently devoted to the mitigation of the Covid-19 outbreak caused by the global spread of the SARS-CoV-2 virus (Zhu et al., 2020). Preventive and health care measurements have been in focus, in addition to the search for potent antiviral and other drugs to deal with the infection and its consequences. A requisite for this is a better understanding of the SARS-CoV-2 itself and the virus-host cell interactions during infection. SARS-CoV-2 is an RNA betacoronavirus that interacts with its cellular receptor ACE2 via its spike protein during the initial step of infection (Lan et al., 2020; Matheson and Lehner, 2020; Walls et al., 2020; Wang et al., 2020; Wrapp et al., 2020). Following binding, the spike protein undergoes cleavage by the transmembrane serine protease 2 (TMPRSS2) that leads to activation of membrane fusion for viral uptake. TMPRSS2 also cleaves ACE2 that is thought to further promote virus entry as shown in detail for SARS-CoV-2 (Hoffmann et al., 2020a).

Angiotensin-converting enzyme 2 is widely expressed in different tissues, including the lung and vascular endothelium. The expression level of ACE2 does not always reflect the clinical course of SARS-CoV-2, suggesting the possibility for additional binding proteins for the virus. Recent data revealed that the spike protein in SARS-CoV-2 is cleaved by the enzyme Furin, unmasking a binding site for neuropilin-1 (NRP1), a receptor at the cell surface (Cantuti-Castelvetri et al., 2020). The Furin cleavage in SARS-CoV-2 is not found in the related SARS-CoV virus. This could explain some of the differences in the behavior and magnitude of the disease caused by these two viruses (Cantuti-Castelvetri et al., 2020; Coutard et al., 2020; Hoffmann et al., 2020b). Mechanistically, it was found that the interaction with NRP1 facilitated the cell uptake of SARS-CoV-2 into cultured cells, suggesting that NRP1 is a crucial factor to potentiate viral infectivity. In line with this, NRP1 is expressed in the nasal epithelium, which is a major target for SARS-CoV-2 to infect people (Cantuti-Castelvetri et al., 2020).

As indicated, the initial steps in the virus-host interactions are crucial for the entry and infectivity of SARS-CoV-2. Blocking the priming or the proteolytic cleavage are possible targets for early drug interventions (Hoffmann et al., 2020b). Recent studies on the host cell membrane and viral interactions have brought novel insights into the role of cellular lipids in the process of viral entry (Carpinteiro et al., 2020; Ripa et al., 2021). Particularly, sphingolipids have been shown to affect cell entry of bacteria and of different viruses (Schneider-Schaulies and Schneider-Schaulies, 2015; Simonis and Schubert-Unkmeir, 2018). In the following we will highlight the novel data obtained with regard to the role of sphingolipids in the biology of SARS-CoV-2, its interactions with the host cell and the potential use of sphingosine 1-phosphate (S1P) as a prognostic marker for COVID-19.

## Sphingolipid Metabolism

Sphingomyelin, and its metabolites comprise an important family of molecules. These molecules function as structural components in the cell membranes, as signaling molecules, and as modulators of enzymatic activity (Pyne et al., 2016; Hannun and Obeid, 2018). The versatility of these molecules has rendered them an extensive and significant role in both physiology and pathophysiology.

**FIGURE 1:**
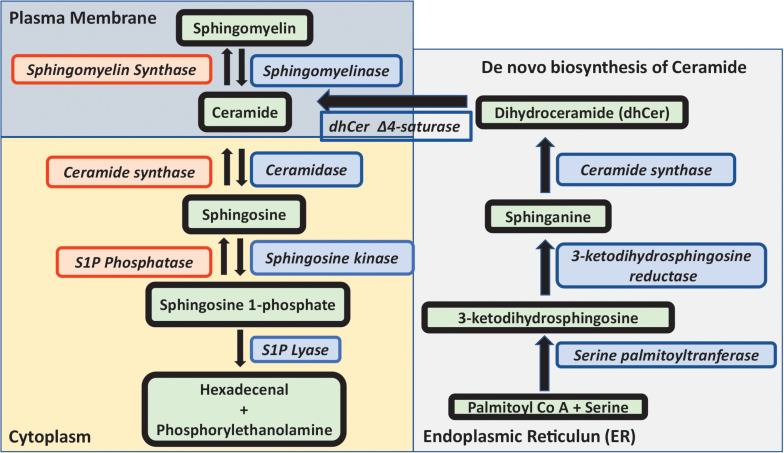
Metabolism of sphingolipids. Outline of sphingolipid metabolism. The *de novo* biosynthesis of ceramide is initiated by the condensation of serine and palmitoyl-Co A to 3-ketodihydrosphingosine, which is reduced to sphinganine, and the addition of an acyl fatty acid to sphinganine forms dihydroceramide which is desaturated to ceramide in the ER. Ceramide is either converted to sphingomyelin in the presence of sphingomyelin synthase or to sphingosine by the action of ceramidase. Sphingosine kinase converts sphingosine to sphingosine 1-phosphate which is either degraded by sphingosine lyase or subsequently converted back to ceramide and sphingomyelin. In this review, sphingosine, ceramide and sphingosine 1-phosphate in relationship to SARS-CoV-2 are discussed in more detail.

The metabolism of sphingomyelin and sphingolipids is extensively complex (Figure 1). Ceramide is the key component in the metabolic flux of sphingolipids and can be formed either by breakdown of sphingomyelin, or through *de novo* synthesis. The latter is initiated by the serine palmitoyltransferase, condensating serine and palmitoyl-coenzyme A to 3-ketodihydrosphingosine. Subsequently, 3-ketodihydrosphingosine reductase reduces 3-ketodihydrosphingosine to sphinganine. The addition of an acyl fatty acid to sphinganine by ceramide synthase results in the formation of dihydroceramide. This is then desaturated by dihydroceramide D4 saturase to ceramide, which takes place in the endoplasmic reticulum (ER). A ceramide-transfer protein transfers ceramide from the ER to Golgi, which is necessary for the synthesis of sphingomyelin. Ceramide can then be converted to several important sphingolipids (Kurz et al., 2019).

The enzyme ceramidase generates sphingosine (2-amino-4-trans-octadecene-1,3-diol) from ceramide, whilst ceramide synthase acts in the opposite direction producing ceramide. Sphingosine can be phosphorylated by sphingosine kinase 1 (SphK1) or sphingosine kinase 2 (SphK2), to produce the active lipid sphingosine 1-phosphate (S1P). S1P in turn is either converted back to sphingosine by the S1P phosphatase, or degraded by the S1P lyase enzyme to hexadecanal and phosphorylethanolamine.

Sphingomyelin is produced from ceramide by sphingomyelin synthase, whereas sphingomyelinase converts sphingomyelin to ceramide. Different forms of sphingomyelinases (SMase) exist, localized to lysosomes (acid SMase), or to the cell surface (neutral SMase). For an in-depth description of the metabolism and functions of different sphingolipids, the reader is referred to several excellent reviews (Gandy and Obeid, 2013; Pyne et al., 2016; Hannun and Obeid, 2018; Kurz et al., 2019; Wigger et al., 2019).

## Sphingosine Influences ACE2 as the Receptor for SARS-CoV-2

Previous studies have shown that sphingosine has antibacterial activities (Bibel et al., 1992; Grassmé et al., 2003; Simonis and Schubert-Unkmeir, 2018; Carstens et al., 2019; Seitz et al., 2019; Verhaegh et al., 2020), and can influence infections by viruses (Grassmé et al., 2005; Miller et al., 2012; Schneider-Schaulies and Schneider-Schaulies, 2015). For SARS-CoV-2 the entry route of infection is usually through the upper respiratory track, raising the question whether sphingosine could have a function in the virus infection. Recent studies employing a model system of epithelial cell cultures and infection with pseudoviral particles carrying the spike protein of SARS-CoV-2 were able to show that sphingosine can interfere with the binding of the SARS-CoV-2 to its receptor ACE2 (Edwards et al., 2020). These experiments included cultures of cells isolated from nasal epithelium of human volunteers, strengthening the relevance of the findings for COVID-2 research. Sphingosine is an amphiphilic molecule, with charges at one end of a saturated fatty acid. The hydrophobic and hydrophilic interactions with ACE2 are likely to contribute to the binding. Sphingosine is relatively insoluble in an aqueous environment, suggesting that it acts from within the membrane. Control experiments using recombinant ACE2 under different conditions indicated that sphingosine directly interacts with ACE2 and not with SARS-CoV-2 or its spike protein. Mechanistically, sphingosine binds to ACE2 at the site used by the spike protein of SARS-CoV-2 and defined by the presence of polar and hydrophobic amino acids (Edwards et al., 2020). However, it is possible that sphingosine may interact with other domains in ACE2 yet to be discovered. Furthermore, these effects described have been obtained by addition of exogenous sphingosine. Several investigations have shown that sphingosine may influence membrane fluidity, and form ordered domains, so called lipid rafts, together with cholesterol. Likewise, sphingosine may interact with other membrane lipids, and this may affect the biophysical properties of the cell membranes (for a comprehensive review on the biophysical properties of sphingosine, see Carreira et al., 2019). Thus, although the study by Edwards et al. (2020) suggested a direct effect of sphingosine with ACE2, the *in vivo* situation may be more complex.

Furthermore, considering the multitude of interactions during the initial priming stage of virus uptake (see above) it would be vital to know whether sphingosine may also influence the activities of TMPRSS2 or Furin and thereby also the NRP1 receptor during the course of infection.

**FIGURE 2:**
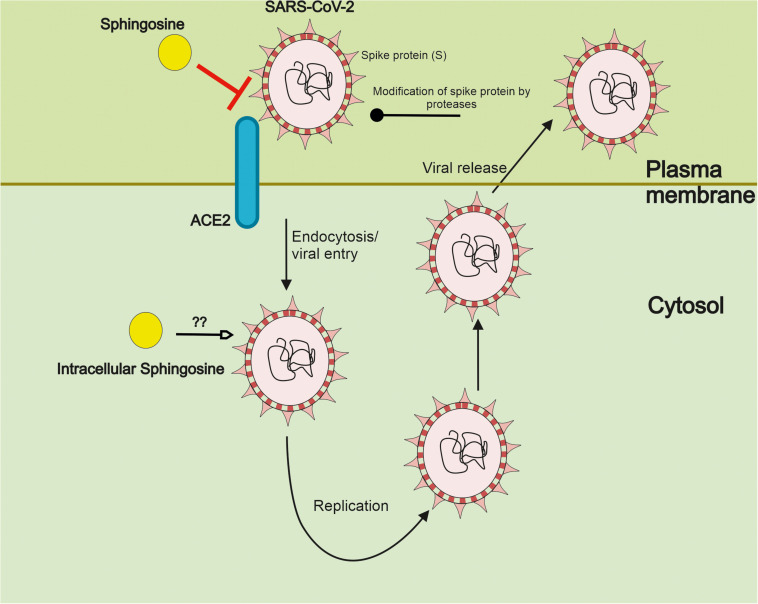
Sphingosine inhibits the interaction of SARS-CoV-2 with its ACE2 receptor. SARS-CoV-2 interacts with its ACE2 receptor on the cell surface via the spike protein (S). Following activity of cell surface protease, the virus is taken up into the cells by membrane fusion events and endocytosis. Subsequent steps in the life cycle of SARS-CoV-2 include viral replication and packaging with the release of new particles to infect further cells. Recent studies employing pseudoviral particles of SARS-CoV-2 indicate that sphingosine can bind ACE2 hindering the interaction of the S protein with the receptor in human epithelial cells. This results in a reduced cell entry and probably infectivity of SARS-CoV-2. Sphingosine and sphingolipids are abundantly present in cell membranes. Sphingosine is here depicted in a schematic way but with the caution that it is not a compound in the classical sense of soluble drug interactions. It remains to be shown whether intracellular sphingosine may influence SARS-CoV-2 at other steps also following viral uptake.

There are also reports showing that sphingosine can interfere with Herpes simplex virus at the level of the lysosome, promoting viral degradation (Lang et al., 2020). It remains to be studied whether sphingosine may similarly act on SARS-CoV-2 further decreasing the infectivity of this virus. Figure 2 gives a summary on the interference by sphingosine of SARS-CoV-2 at the level of the cell surface and possibly intracellularly.

Adding to this, sphingosine itself can be modified by different enzymes including phosphorylation that influence its concentration at the cell surface (Figure 1). As discussed below the serum levels of S1P are altered during the course of COVID-19 disease underscoring the importance of sphingosine in the SARS-CoV-2 infection. It is worth mentioning here that sphingosine and S1P have opposite effects in cells, S1P being a pro-survival molecule, whereas sphingosine is a pro-apoptotic molecule (Pyne et al., 2016). Thus, the relative concentrations of these in cells or in the serum may have different effects.

## Ceramide in the Regulation of SARS-CoV-2

Sphingomyelin is a major sphingolipid in cell membranes. Ceramide is cleaved from sphingomyelin by the action of the enzyme, Acid sphingomyelinase (ASM) present in lysosomes and cell membrane (Figure 1). The ASM/ceramide system is involved in host defense reactions (Coant et al., 2017; Simonis and Schubert-Unkmeir, 2018; Prakash et al., 2021). Ceramide is also associated with metabolic diseases in humans, including obesity (Chaurasia and Summers, 2021). The possible links between deregulated ceramide metabolism in human metabolic diseases and the likelihood for a poor outcome following a COVID-19 infection are so far not fully understood.

**FIGURE 3:**
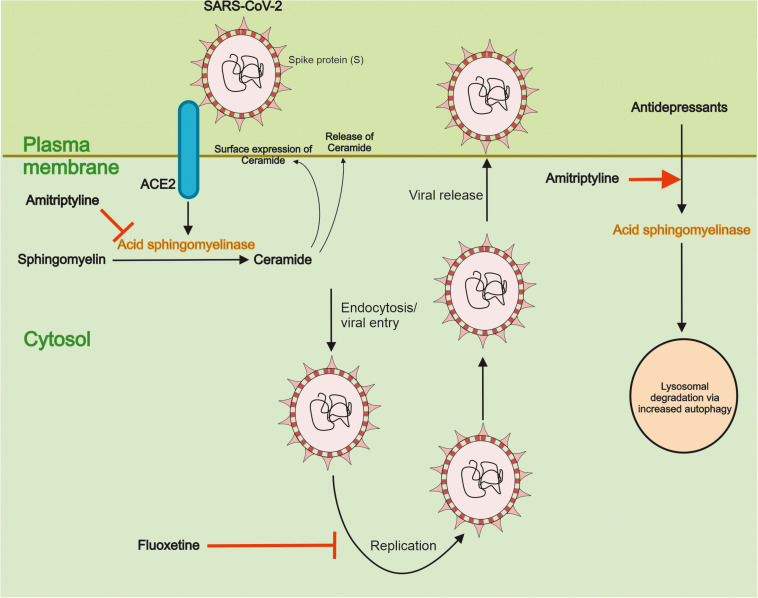
Functional interactions between SARS-CoV-2 and the ASM/ceramide system. Effects of antidepressant drugs. Ceramide plays an important role in signaling by different cell surface receptors. Ceramide is produced from sphingomyelin by the action of Acid sphingomyelinase (ASM) in lysosomes and cell membranes. It was recently shown that the interaction of SARS-CoV-2 with ACE2 activates ASM in cells and induce an accumulation of ceramide at the cell surface. The antidepressant drug Amitriptyline reduced ASM levels and activity, and the production and accumulation of ceramide. This in turn diminished the uptake of SARS-CoV-2 in human epithelial cells. Amitriptyline further prevented the infection of nasal epithelia cells by SARS-CoV-2 in an *ex vivo* model using human volunteers (see text for details). The antidepressants act by functionally inhibiting ASM and reducing ceramide levels in membranes and they are also known to influence autophagy. Fluoxetine was further shown to inhibit the SARS-CoV-2 replication through effects on endolysosome acidification and cholesterol levels. More detail studies are, however, warranted for how different antidepressants and other drugs may affect the viral uptake, replication and the other intracellular steps in the life cycle of SARS-CoV-2. Likewise, clinical studies are important to conduct with the antidepressant and other promising drugs with regard to their potential use and safety for treatment of patients afflicted by COVID-19.

Recent studies using pseudoviral particles of SARS-CoV-2 spike protein for infection revealed that the virus can activate ASM and induce the accumulation of ceramide in human epithelial cells (Carpinteiro et al., 2020). In contrast, reducing ceramide at the cell surface using antibodies or using the degrading enzyme, ceramidase, inhibited the infection of nasal epithelial cells by the SARS-CoV-2 pseudovirus. Most importantly, a functional inhibition of ASM by the antidepressant drug, amitriptyline decreased ceramide levels and prevented the infection of the cells by the viral particles. Amitriptyline also diminished the infection by the *bona fide* SARS-CoV-2 virus as shown in the human epithelial cells (Carpinteiro et al., 2020). Amitriptyline is used in the clinics to treat symptoms of major depression. In an attempt to study its action in an *ex vivo* setting, nasal epithelial cells were collected from volunteers having received a low dose of the drug followed by infection of the cells by the SARS-CoV-2 S pseudovirus particles. Remarkably enough, data showed that a single dose of amitriptyline given up to 24 h before, prevented viral infections of the isolated cells and this was associated with a decrease in the activity of ASM (Carpinteiro et al., 2020). Together these findings demonstrate that the activity of ASM, and the corresponding changes in ceramide, crucially influence the infection ability of SARS-CoV-2, as studied using *in vitro* and *ex vivo* systems. These results also raise pertinent questions regarding the mechanisms underlying the interplay between SARS-CoV-2 and the ASM/ceramide system in cultured cells and *in vivo*. A schematic view on the relationships between ASM/ceramide and of SARS-CoV-2 is provided in Figure 3.

Ceramide can interact with several proteins in the cell including phospholipase A2, cathepsin D, and protein kinase C isoforms (discussed in Carpinteiro et al., 2020). Functionally it is thought that ceramide influence receptor signaling via formation of ceramide-enriched membrane domains, so called lipid rafts, at the cell surface. Ceramide also play a role in insulin signaling (Wigger et al., 2021) and in metabolic diseases in humans, including obesity (Chaurasia and Summers, 2021). This is interesting as co-morbidities like obesity and type 2 diabetes are associated with a worse outcome in COVID-19. In view of this, more detailed studied on the relationships between ceramide and SARS-CoV-2 are worthwhile pursuing.

## Antidepressant Drugs and COVID-19

As indicated above amitriptyline, prevented the uptake of SARS-CoV-2 in model systems of human epithelial cells (Carpinteiro et al., 2020). The mode of action of amitriptyline was linked to the functional inhibition of ASM decreasing the surface concentrations of ceramide produced from sphingomyelin. Mechanistically, amitriptyline accumulates in the lysosomes and in acidic sub-compartments in cell membranes, and can thereby influence the localization of ASM, leading to its degradation. Other antidepressants also affect the ASM/ceramide system in cells (Gulbins et al., 2013; Breiden and Sandhoff, 2019). In line with this, drugs like escitalopram, desipramine, imipramine, sertraline, and fluoxetine also prevented infection of cultured epithelial cells by the pseudovirus particles of SARS-CoV-2 (Carpinteiro et al., 2020). These findings support the view that the ASM/ceramide system is an important common target for the action of antidepressants in COVID-19. Moreover, fluoxetine can inhibit the replication of SARS-CoV-2 (Figure 3), suggesting that this drug may have a dual function, preventing cell uptake, and viral replication (Zimniak et al., 2021).

In another study, fluoxetine diminished the acidification in the endolysosomal compartment and increased endolysosomal cholesterol content, inhibiting SARS-CoV-2 infection in human bronchioepithelial cell lines (Schloer et al., 2020). Similar effects were observed with two other drugs, amiodarone and imipramine. It was inferred that, fluoxetine could possibly affect the distribution of cholesterol between endolysosomal compartments and other membranes such as; plasma membrane and ER-Golgi interface, inhibiting the early stages of SARS-CoV-2 entry into cells. The resulting increase in endolysosomal cholesterol content can lead to dysregulation of the vacuolar-type membrane (v-ATPase) pumps that help maintaining the luminal pH and in turn affecting the pH-dependent proteases.

Collectively, these findings show that antidepressants can interfere with different steps in the life cycle of SARS-CoV-2 that needs to be explored further (Figure 3).

The concept of drug interaction with virus entry and replication may be significant for other drugs too. Based upon the physiochemical (cationic amphiphilic) properties of these substances it was proposed that several drugs used in psychiatry as well as some antihistamines could have protective effects against SARS-CoV-2 infections (Gitahy Falcao Faria et al., 2021). On the other hand, the use of antipsychotic drugs also has caveats as these drugs have side-effects and may cause intoxications as shown recently for clozapine in COVID-19 (Tio et al., 2021). Caution has to be exerted regarding their use as off-label treatments, and the actual dose given to COVID-19 patients (Anmella et al., 2020).

Antidepressant, and antipsychotic drugs have different mechanisms of actions, including effects on cell stress responses and autophagy (Srinivasan et al., 2020). As shown previously, antidepressants influence autophagy via ceramide (Gulbins et al., 2018) but also other mechanisms that may be relevant for our understanding of SARS-CoV-2 pathogenesis and therapy. The physiological significance of the autophagy pathway, particularly in neuronal cells in relationship to overall sphingolipid metabolism and the drug action remains to be explored.

Antidepressants, such as selective serotonin and noradrenalin reuptake inhibitors (SSRI and SNRI), are frequently used in the adult population to treat depression and anxiety. They are generally well-tolerated and can also be used in low doses and short-term. A recent meta-analysis showed that treatment with antidepressants can alter peripheral cytokines and chemokines in patients with major depressive disorder (Köhler et al., 2018). Thus, the levels of IL-10, TNF-alpha, CCl-2, and IL-6 were decreased in patients receiving the drug. As known, these cytokines are among those that are elevated in SARS-CoV-2 infected patients, suggesting a possible beneficial role of antidepressants in the viral infection. It was recently reported studying a large number of patients hospitalized for COVID-19 that the ongoing use of antidepressant drugs significantly reduced the risk of intubation or death (Hoertel et al., 2021). The results obtained might related to a direct anti-inflammatory and anti-viral effects of the drugs with their actions on acid sphingomyelinase activity. However, detailed information is required about the actions of different antidepressants, drug doses, duration of treatment and ongoing versus previous drug use in COVID-19 patient to be able to draw more definitive conclusions.

The work by Hoertel et al. (2021) was an observational study that should be followed-up by double-blind controlled randomized clinical trials. Large-scale register studies on other cohorts would also be helpful to further elucidate whether the use of antidepressant and/or other psychoactive drugs is associated with lower prevalence of COVID-19. In this instance questions about the long-term effects and complications of COVID-19 including prolonged fatigue, cognitive impairments, anxiety and psychological well-being are thereby relevant issues to be considered.

In conclusion, available data supports the view that antidepressant can interfere with the entry of SARS-CoV-2 in cells and reduce inflammation markers that are associated with the viral infection. These could add to the action of these drugs in alleviating the pathophysiological consequences of SARS-CoV-2. Currently trials are on-going for several drug candidates (Blaess et al., 2021), including some with psychoactive substances like clomipramine (Nobile et al., 2021). However, more detailed clinical studies are required to validate their usefulness as potential drugs against viral infections including COVID-19 in large study populations. Along with this, crucial issues to be considered are the safety of the psychotropic drugs and their interactions with other medications when administered to COVID-19 patients afflicted by other diseases (Ostuzzi et al., 2020).

## S1P as a Prognostic Marker for SARS-CoV-2

Recent studies indicate that levels of S1P in patient serum change during the course of COVID-19 with lower circulating S1P observed in patients with a more severe clinical course (Marfia et al., 2021; Rosen and Oldstone, 2021). In view of this, it was suggested that S1P could be a prognostic marker to define the outcome of COVID-19. S1P serum levels were also decreased in patients suffering from acute respiratory distress syndrome (ARDS), which is a feared complication of SARS-CoV-2 (Zhao et al., 2020). The reported findings are based upon a rather small number of patients and need to be confirmed in future investigations using larger study cohorts.

Sphingosine-1-phosphate is a signaling molecule with a variety of actions acting via its specific G protein-coupled receptors (S1P receptors 1–5). S1P exerts several biological processes including regulation of cell signaling, cell death, proliferation, and cell migration (Pyne et al., 2016). These effects are complex and occur in an interplay with other factors. Especially in the immune system S1P influences the migration of immune cells that may enhance a pro-inflammatory response (Chiricozzi et al., 2018). The level of S1P in cells is tightly regulated but can be altered in different disease conditions. In the blood S1P is mainly in erythrocytes and platelets as well as in the endothelial cells of the vasculature. It is thought that S1P is important for the protection of vascular integrity and the endothelial barrier that is disrupted in lungs during the course of COVID-19 and in ARDS (Pedersen and Ho, 2020). According to previous studies, chemical compounds acting on S1P receptors can dampen the pro-inflammatory immune response and counteract bacterial infections. Similarly, S1P analogs can provide protection against pathophysiological effects of influenza virus and increase the survival of infected mice (Naz and Arish, 2020). With regard to COVID-19 clinical trials are currently underway to examine the efficacy of S1P receptor modulators and chemical compounds in alleviating SARS-CoV-2 infection (McGowan et al., 2020). Fingolimod (also named FTY720) is a compound taken up by the cells, then phosphorylated predominantly by SphK2, and transported out of the cell. The phosphorylated form binds to S1P receptors, and evokes an internalization of the receptors. This leads to a desensitization and attenuation of the signaling pathways activated by S1P. FTY720 can further target four of the five S1P receptors and is in treatment of multiple sclerosis (for a review, see Chun et al., 2021). The multitude of receptors and downstream signals can make the prediction of the final biological outcome unclear. Compounds with higher selectivity for specific S1P receptors or interfering with the phosphorylation step of sphingosine to S1P may provide some clinical benefits as potential treatments.

Given that sphingosine interacts with ACE2 and prevent SARS-CoVid-2 cell entry, S1P derived from sphingosine may also influence this early step of the infection. An increasing number of studies have shown changes in the immune profile of patients with COVID-19 with some of them linked to the severity of the disease (Laing et al., 2020). In particular, increases in cytokines, such as interleukin-6, are correlated with the cytokine storm and the intense inflammatory condition occurring during COVID-19 (Pedersen and Ho, 2020). How the reduction in S1P may alter the immune markers at different stages of the disease is an important question to pursue. In conclusion, data on low circulating S1P levels in SARS-CoV-2 infections lends credence to the view that S1P and its signaling deficiency may play a role in the disease course. Clinical cohorts including large number of patients are required to define whether S1P could be a potential biomarker or therapy target in COVID-2.

## Future Perspectives

Recently a thorough review of the possibilities to repurpose sphingolipid compounds, especially those affecting the S1P/S1PR signaling pathway, has been published (McGowan et al., 2020). An important action of these compounds, including the above mentioned FTY720, is to target the hyperinflammation (cytokine storm) and multiorgan failure that accompany COVID-19 infections by preserving the integrity of the vasculature and preventing thrombotic complications. Sphingolipids have several beneficial roles in combating viral diseases including their long-term effects. In view of this more clinical and well-designed trials using compounds and drugs affecting sphingolipid metabolism are vital to pursue further for SARS-CoV-2.

As discussed in this review, sphingolipids as lipid molecules can affect SARS-CoV-2 and its cell biology in at least two ways; by binding of SARS-CoV-2 to ACE2 receptors, and by means of changing levels of ceramide. The latter is directly amenable to drug interventions, as several antidepressants are known to reduce ASM levels, thus diminishing the production of ceramides. Antidepressants are widely used in the clinics and considered to have a good safety profile. This emphasizes the need to perform well-designed clinical trials with different antidepressants to clarify their possible role in antagonizing SARS-CoV-2 infection and in treatment of COVID-19. Promising avenues to pursue include also the possibility that S1P is a biomarker for the severity of the disease, and that an enhancement of S1P receptor signaling may have beneficial effects for immune responses during COVID-19. Results of studies on these issues may in the long-run benefit further drug development and treatment of COVID-19 and possible also of other viral diseases.

## Author Contributions

KT and DL designed the project. All authors contributed to the article and approved it for publication.

## Conflict of Interest

The authors declare that the research was conducted in the absence of any commercial or financial relationships that could be construed as a potential conflict of interest.
